# Risks and Benefits of Thrombolytic, Antiplatelet, and Anticoagulant Therapies for ST Segment Elevation Myocardial Infarction: Systematic Review

**DOI:** 10.1155/2014/416253

**Published:** 2014-02-06

**Authors:** Bruno Ramos Nascimento, Marcos Roberto de Sousa, Fábio Nogueira Demarqui, Antonio Luiz Pinho Ribeiro

**Affiliations:** ^1^Serviço de Cardiologia e Cirurgia Cardiovascular, Hospital das Clínicas da Universidade Federal de Minas Gerais, Avenida Professor Alfredo Balena 110, Campus Saúde, 30130-100 Belo Horizonte, MG, Brazil; ^2^Serviço de Hemodinâmca do Hospital Universitário São José (INCOR Minas), Rua Aimorés 2896, Barro Preto, 30140-073 Belo Horizonte, MG, Brazil; ^3^Departamento de Clínica Médica da Faculdade de Medicina da Universidade Federal de Minas Gerais, Avenida Professor Alfredo Balena 190, Campus Saúde, 30130-100 Belo Horizonte, MG, Brazil; ^4^Departamento de Estatística, Instituto de Ciências Exatas, Universidade Federal de Minas Gerais, Avenida Antônio Carlos 6.627, Campus Pampulha, 31270-901 Belo Horizonte, MG, Brazil

## Abstract

*Objectives*. Assess the impact of associating thrombolytics, anticoagulants, antiplatelets, and primary angioplasty (PA) on death, reinfarction (AMI), and major bleeding (MB) in STEMI therapy. *Methods*. Medline search was performed to identify randomized trials comparing these classes in STEMI treatment, at least 500 patients, providing death, AMI, and MB rates. Similar arms were grouped. Correlation between number of drugs and PA and the outcomes was evaluated, as well as correlation between the year of the study and the outcomes. *Results*. Fifty-nine papers remained after exclusions. 404.556 patients were divided into 35 groups of arms. There was correlation between the number of drugs and rates of death (*r* = −0.466, *P* = 0.005) and MB (*r* = 0.403, *P* = 0.016), confirmed by multivariate regression. This model also showed that PA is associated with lower mortality and increased MB. Year and period of publication correlated with the outcomes: death (*r* = −0.380, *P* < 0.001), MB (*r* = 0.212, *P* = 0.014), and AMI (*r* = −0.231, *P* = 0.009). *Conclusion*. The increasing complexity of STEMI treatment has resulted in significant reduction in mortality along with increased rates of MB. Overall, however, the benefits of treatment outweigh the associated risks of MB.

## 1. Introduction

Approximately 1.7 million people are hospitalized annually in the United States of America due to acute coronary syndromes (ACS), with almost one-quarter of cases showing ST-elevation acute myocardial infarction (STEMI) [1]. Its physiopathology is closely related to thrombus formation (after a complex process that culminates in platelet adhesion, activation and formation of platelet cluster) and activation of the coagulation cascade, over unstable atherosclerotic plaques [2, 3]. With the evolution of STEMI treatment, drugs acting on these mechanisms: antiplatelet, anticoagulant, and thrombolytic therapies have been extensively tested in numerous studies, in different doses and associations, often in conjunction with interventional techniques, with significant reduction of morbi-mortality. However, there was also a marked increase in major bleeding rates, that is, difficult to measure objectively due to the lack of a standardized methodology for this purpose [4]. Besides this, drug associations not adequately tested in large trials have been incorporated by international guidelines [5–7].

Given the broad spectrum of clinical outcomes (with different definitions) and the heterogeneous criteria for adverse events (specially bleeding) reported in the numerous published studies, it's very difficult to estimate the real clinical benefit of each drug class for STEMI treatment. Pooled analysis of a significant number of studies is necessary, taking into account all the heterogeneity among them, in order to quantitatively evaluate the overall risk and benefit of the progressive addition of of drugs over time. A systematic review with quantitative approach may be a useful tool for this purpose, with its ability to incorporate the methodological issues observed [8, 9].

## 2. Objectives

To evaluate, by systematic review with quantitative analysis, the impact of the progressive addition of drug classes (thrombolytics, anticoagulants, and antiplatelets) and primary angioplasty (PA) on death, reinfarction (AMI), and major bleeding (MB) rates in patients with STEMI.

## 3. Materials and Methods

Initially, a search was performed in Medline and Cochrane databases looking for articles with similar objectives and methodology. No similar publications were found.

A systematic medline search was performed using the MeSH terms: *Acute [All Fields] AND myocardial infarction/therapy [Mesh Terms] AND ((“1”[EDAT]: 2009/12/31[EDAT]) AND humans [MeSH Terms] AND (English[lang] OR Spanish[lang] OR Portuguese[lang]) AND Randomized Controlled Trial[ptyp] AND adult[MeSH Terms]),* to identify randomized trials in English and Spanish that compared drugs and interventional techniques for the acute treatment of STEMI.

Paper selection criteria were (1) randomized trials involving human adults and (2) comparing drugs in the treatment of STEMI: thrombolytics, antiplatelets, and anticoagulants. Studies comparing thrombolytics and PA were included. (3) Sample size: minimum 500 patients (defined to reduce inaccuracies in adverse events estimates and bias derived from published trials with reduced quality and power [10]) and (4) death, AMI and MB rates in the time frame closest to 30 days adequately provided.

The following aspects were observed: (a) defined inclusion criteria, sample selection explained, adequate description of diagnostic criteria, clinical and demographic characteristics and inclusion of all eligible patients; (b) objectives and results presented without explicit bias; (c) clinical variables clearly defined, including technical details of the treatments employed, clear definition of adverse cardiovascular events, and the bleeding criteria; (d) adequate statistical analysis with adjustment for all important factors.

Exclusion criteria were (1) nonrandomized studies; (2) studies with different scopes; (3) studies comparing interventions other than those mentioned, (4) studies involving only non-ST elevation acute coronary syndromes.

Article selection was independently done by two researchers (BRN and MRS), in three stages: (a) exclusion by title, (b) exclusion by abstract, and (c) exclusion by full text reading. Discrepancies were solved in consensus. Inclusion of additional data, provided by authors or contributors, was allowed as well as evaluation of related articles, editorials, and subanalyses.

After the final selection, demographic methodological, and clinical data were tabulated. Clinical outcomes and bleeding criteria were tabulated according to the trial's definitions. In some articles, when bleeding criteria were not explicit, we considered criteria previously reported in relevant publications.

Study arms were grouped by similarity, according to the association of therapeutic classes: aspirin (ASA), thienopyridines (TP), unfractionated heparin (UFH), low molecular weight heparin (LMWH), glycoprotein IIbIIIa (GPI), direct thrombin inhibitors (DTI), non fibrin-specific thrombolytics (streptokinase and urokinase-KS), fibrin-specific thrombolytics (FS), and PA. The class was considered when, in the arm, at least 50% of patients had received it. A regrouping was also carried out (“pure” groups), and the classes were considered when at least 85% of patients received them, with maximum contamination of 30% of drugs that were not part of the group's therapeutic regimen.

## 4. Statistical Analysis

We performed indirect comparisons between similar study arms [11]. Using the software *Statistical Package for Social Sciences* (SPSS) version 16.0.1 (IBM, Copyright 2007) a correlation (Spearman's test) between the number of drug classes and the clinical outcomes (death, AMI, and MB) was performed. We also tested the correlation between the year of the study's publication and the outcomes of interest. For this analysis, we considered the year of publication, and we also grouped the trials into five time periods: (a) up to 1989; (b) 1990–1994; (c) 1995–1999; (d) 2000–2004; (e) 2005–2009. The outcomes of the groups of study arms submitted to PA were compared with those not submitted by means of nonparametric tests. All the analysis was also performed for the “pure” groups. Additionally, a binomial multivariate regression model was adjusted, considering the number of classes used and PA as independent variables and death, AMI, and MB as outcomes, with the SAS/STAT Software, version 9.2 (*Copyright SAS Institute Inc., Gary, NC, USA*). A latent variable was inserted in this model to outweight the heterogeneity between trials, and the arms were weighted by sample size. A *P* value of <0.05 was considered to be statistically significant.

## 5. Results

**Figure 1:**
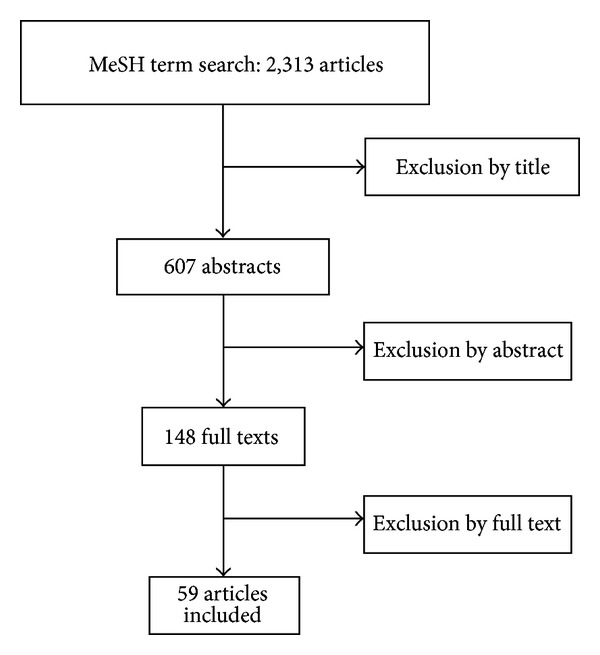
Flowchart of article selection by peer review.

The search initially returned 2,313 articles. After the initial exclusion, 607 abstracts were selected, and 148 articles remained for full text reading, after which 59 articles were included in the final database (Figure 1), totaling 133 treatment arms. The publication period was from 1985 to 2009.

The total sample size was 404,556 patients, with average age of 61.0 years: 39.9% were hypertensive, 15.0% diabetic, 47.3% were smokers, and 14.2% had a history of prior AMI. Regarding treatment, 89.8% received ASA, 20.4% underwent PA, 25.0% received TP, 73.4% received UFH, 8.0% received LMWH, 16.1% received GPI, 4.5% received DTI, and 71.2% received thrombolytics (47.0% FS and 27.2% FS). The weighted average follow-up time was 23.3 days. The arms were grouped by similarity in 35 groups, according to the described definition (Table 1 [12–71]).

Nineteen different major bleeding criteria were identified. The most used was *Thrombolysis In Myocardial Infarction* (TIMI [72]) criteria, followed by the need for any transfusion, the *Global Use of Strategies to Open Occluded Coronary Arteries* (GUSTO [60]) and the modified GUSTO criteria (excluding hemorrhagic stroke), present in 21.1%, 18.8%, 18.0%, and 8.1% of the study arms, respectively. There was a wide variability in the clinical and laboratorial definitions for the different bleeding criteria found, and their severity diverged considerably (Table 2 [60, 72]).

### 5.1. Correlation: Number of Drugs and Primary Angioplasty and Adverse Events

**Figure 2:**
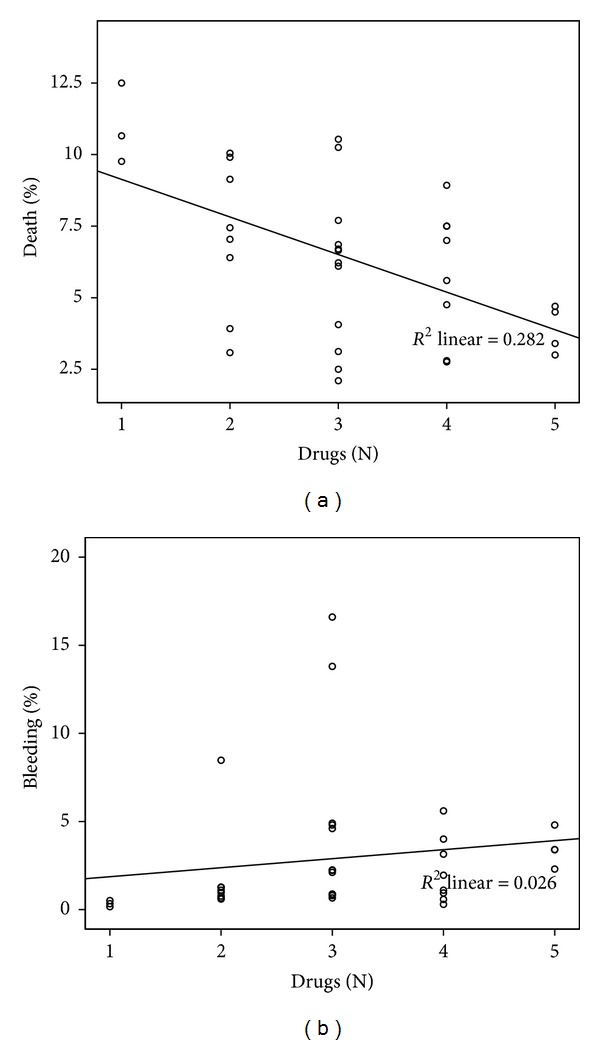
Correlation between number of classes of drugs and (a) mortality (*r* = −0.466, *P* = 0.005) and between number of classes of drugs and (b) major bleeding (*r* = 0.403, *P* = 0.016).

Considering the 35 therapy groups, there was a statistically significant correlation between the number of drugs used and the rates of death (*r* = −0.466, *P* = 0.005) and MB (*r* = 0.403, *P* = 0.016) (Figure 2) without correlation with AMI (*r* = −0.034, *P* = 0.847). Graphically, the mortality line is steeper compared to the bleeding line in these correlations.

Similarly, comparing the proportions of these outcomes between the groups in which PA was performed with the groups not submitted to intervention, the first had significantly lower mortality (3.98 ± 1.81 × 7.56 ± 2.45, *P* = 0.001) and a higher rate of MB (5.71 ± 5.22 × 1.64 ± 1.46, *P* = 0.006), with similar AMI rates (*P* = 0.286). The findings were similar for the “pure” arms.

### 5.2. Multivariate Regression Model

The adjusted multivariate regression model showed negative association between the number of drugs used and mortality (*β* = −0.233, *P* < 0.001) and positive association between this number and the rates of MB (*β* = 0.405, *P* = 0.012). In addition, PA also had negative association with death (*β* = −1.605, *P* < 0.001) and positive association with MB (*β* = 2.251, *P* = 0.039). This data confirms the correlation's findings. However, in the presence of PA, the *odds* ratio for mortality reduction by the addition of one drug class loses significance, suggesting additional benefit of intervention. Similarly in the MB model, although there is association between the number of drugs and PA with this outcome, in patients undergoing PA the effect of the addition of a drug class on MB rates loses significance. In all models, the effect of the latent variable was significant, meaning significant heterogeneity between trials (Table 3).

The outcome AMI did not have significant association with the number of drugs (*β* = 0.036, *P* = 0.550) and the performance of PA (*β* = 0.629, *P* = 0.197) similarly to the correlation models.

### 5.3. Correlation: Year and Period of Publication and Adverse Events

**Figure 3:**
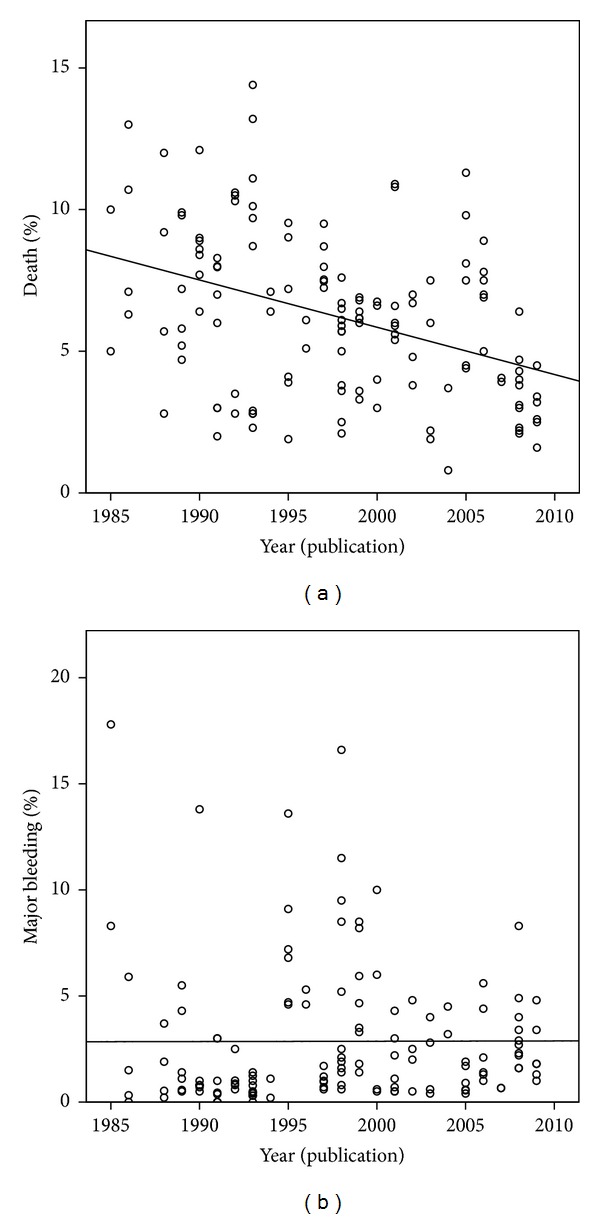
Correlation between year of publication and (a) mortality (*r* = −0.380, *P* < 0.001) and between year of publication and (b) major bleeding (*r* = 0.212, *P* = 0.014).

The year of publication had a statistically significant correlation with the three outcomes assessed: death (*r* = −0.380,*P* < 0.001), AMI (*r* = −0.231, *P* = 0.009), and MB (*r* = 0.212, *P* = 0.014) (Figure 3). Similarly, the five periods in which the publication years were grouped (weight = 1 for every five years) had a statistically significant correlation with the rates of these three outcomes (resp.,  *r* = −0.325, *P* < 0.001, *r* = −0.236, *P* = 0.007, *r* = 0.214, *P* = 0.013). Considering only the 21 groups of “pure” arms, the correlation remained statistically significant for death (*r* = −0.272, *P* = 0.010) and AMI (*r* = −0.366, *P* = 0.001) but not for MB.

## 6. Discussion

Since the first studies involving the treatment of STEMI, there has been debate about the “risk versus benefit” of prescribing drugs that are potentially related to coagulation. In the first large-scale studies [49, 73, 74], the measurement of this net benefit was simpler, since the reduced number of classes involved made individual effects on major adverse events easier to evaluate. Besides this, considering that STEMI was a disease practically without specific treatment, the clinical benefit of introducing the first antiplatelets and reperfusion strategies [49, 73–78] was clearly relevant.

As treatment regimens became more complex, drug interaction started to play a role in this scenario and so did the large variability of doses tested (and their adjustment to specific populations), making it progressively more difficult to compare drug classes. We opted to perform indirect comparisons as a way to estimate numerically the effect of adding therapies, by grouping heterogeneous arms of studies, covering all STEMI pharmacological treatment periods [9, 79, 80]. Methodologically, a reliable estimate of the effect may be obtained when direct comparisons are not feasible, even considering the loss of randomization [11, 81].

The correlation between number of drugs used and the reduction of mortality and the correlation between this outcome and the year/period of publication of the study are in accordance with previously published data [82]. Graphically, there seems to be a trend towards greater reduction of mortality rates compared to MB (Figures 2 and 3), indirectly suggesting that the clinical benefit may still be overlapping the increasing bleeding risk. This balance, however, is probably close to its limit. The significantly lower mortality in the groups submitted to PA confirms its well-known additional benefit that might hide a possible near-equivalence between mortality reduction and increased bleeding associated with thrombolysis. This hypothesis is reinforced by a significant loss of interaction between the number of drugs and mortality in the presence of PA.

Although there are specific recommendations for the definition of bleeding events [83], we observed a large number of criteria, some of which developed for specific trials (Table 2), often with prognostic accuracy not well established [84]. Despite this heterogeneity, a considerable number of studies used two of the most accepted criteria—TIMI and GUSTO [60, 72]: the first is essentially based on laboratory data, while the second relies on clinical observations and seems to have a greater prognostic value [85]. However, it does not appear to be a trend towards standardization in the most recent large-scale trials [20, 86, 87]. This lack of standardization, besides making it more difficult to compare the safety of therapeutic strategies, raises critical questions about the possible adjustment of specific criteria, aiming to favor the results of a drug class under evaluation.

There's a temporal trend towards increased rates of MB, related to the increasing complexity of therapies (number of drugs), currently averaging around 3-4% (Figures 2 and 3). Given the known relationship of bleeding complications with prognosis [84, 88], we must be aware of this fact, since some associations recommended by guidelines [5–7] were systematically tested only in separate [23, 39, 45, 56]. Considering that part of the cumulative benefit over mortality may be related to the advent of PA (as pointed out by the the regression model), in the arms that used thombolytics—agents with potential bleeding effect—there, may be a trend to nullify the net benefit. On the other hand, this hypothesis is counterbalanced by the observed association between PA and increased MB: in its presence, the effect of adding one class of drug on bleeding complications becomes not significant. One must also consider that PA became a routine procedure when more aggressive antiplatelet and anticoagulant strategies were already established.

Previously published data showed significant bias related to the risk profile of randomized and nonrandomized patients in large studies, besides the fact that the inclusion of unpublished studies may improve the accuracy of risk estimates [89, 90]. The compilation of a large volume of data, including studies with a considerable sample size (>500 patients), may dilute these biases, but they should be considered when analyzing the external validity of the review.

Despite these considerations, the trend towards mortality reduction and increased MB as drugs were added over time are plausible and consistently demonstrated by two different analytic models. This review highlights the need for standardization of the methodology for assessing MB complications in randomized trials, as recommended by the literature, in order to make possible accurate inferences about the risk/benefit profile of drug associations.

## 7. Limitations

The main limitations of this analysis are related to the methodology of systematic reviews and metaanalysis with indirect comparisons itself. Qualitatively, one should note the different eras of clinical research covered by the publications (1985 to 2009), which itself accounts for the great methodological discrepancies, related to sample selection and allocation, study design, definitions of outcomes and clinical endpoints and follow-up strategies. Besides that, the results may also be influenced by the wide variability of MB definitions and the great heterogeneity of drug associations, doses, and regimens. Similarly, the techniques and results of PA have significantly improved over the decades covered by this review. Such differences make secondary data analysis even more susceptible to bias and the direct comparison between groups methodologically impossible.

For this reason, indirect comparisons were performed between groups. This methodology can be applied when arms of studies with different therapeutic schemes are found. At the cost of loss of the randomization effect, with this approach it is possible to obtain an estimate without treatment effect bias, even if treatment and control arms differ in their baseline characteristics, as observed in this heterogeneous sample of trials [11, 81]. However, in the adjusted models, a weight = 1 was attributed to all drug classes, which must be carefully interpreted, since the benefit/harm profile of the drugs may vary and cannot be precisely estimated. Thus, no extrapolations can be made about the effect of each specific class nor about the odds for adverse events in a specific therapeutic scheme.

## 8. Conclusion

This systematic review with quantitative analysis demonstrated a negative association between the number of classes of drugs used for the acute treatment of STEMI and mortality rates and a positive association with MB. From the perspective of the main trials involving STEMI treatment, the optimal therapy recommended by guidelines is in the boundary of clinical benefit, with mortality rates currently around 5%, at the expense of increasing bleeding complications, apparently of lesser magnitude up to this point. Our findings depict numerically subjective observations: it seems that the net clinical benefit still outweighs the bleeding risk. Additional studies and metaanalysis with individual associations are necessary to clearly estimate the benefit of specific therapies, especially in complex associations.

## Figures and Tables

**Table 1:** Groups of treatment regimens (grouping of similar arms) and pure groups.

Number	Regimen	Studies (references)	Number of arms	N	Number of “pure” arms	N in “pure” arms
1	ASA + PA + TP + UFH + GPI	[12–19]	13	9,369	10	4,997
2	ASA + PA + TP + DTI	[20]	1	1,800	1	1,800
3	ASA + PA + TP + UFH + GPI + FS	[21]	1	298	1	298
4	ASA + TP + UFH + GPI + FS	[21]	1	300	1	300
5	ASA + UFH + FS	[22–43]	38	104,754	33	82,283
6	ASA + UFH + GPI + FS	[22]	1	8,328	1	8,328
7	ASA + PA + GPI	[44]	1	2,885	—	—
8	ASA + PA + TP + GPI	[44]	1	2,860	—	—
9	ASA + LMWH + FS	[23, 45]	2	12,296	1	2,040
10	ASA + PA + TP + UFH	[12, 14, 15, 46, 47]	5	2,647	3	1,922
11	ASA + TP + LMWH + FS	[45]	1	818	—	—
12	ASA + TP + UFH + FS	[39, 45, 46]	3	2,992	—	—
13	ASA + LMWH + KS	[48]	1	253	1	253
14	ASA + KS	[30, 48–53]	7	29,824	4	3,428
15	ASA + DTI + KS	[54, 55]	2	9,119	2	9,119
16	ASA + UFH + KS	[30, 32, 36, 37, 40, 55–60]	15	75,382	13	46,292
17	ASA + PA + UFH	[27, 61]	3	826	2	584
18	ASA + FS	[30, 41, 52, 53, 62]	7	38,298	2	1,471
19	ASA + UFH + FS + KS	[40, 60]	2	10,471	2	10,471
20	ASA + PA + UFH + GPI	[61]	1	241	1	241
21	ASA + DTI + FS	[34]	1	1,511	—	—
22	TP + KS	[51]	1	450	1	450
23	UFH + FS	[63, 64]	2	3,136	2	3,136
24	UFH	[63–65]	3	4,200	3	4,200
25	ASA + UFH	[38, 42, 58, 59]	4	4,387	3	1,512
26	ASA	[49, 50]	2	10,854	1	2,259
27	ASA + PA + UFH + FS	[66]	1	195	—	—
28	ASA + TP + LMWH + GPI + FS	[67]	1	522	—	—
29	ASA + PA + TP + LMWH + GPI + FS	[67]	1	537	—	—
30	ASA + TP + UFH + KS	[56]	1	22,961	—	—
31	ASA + TP + KS	[68, 69]	2	13,846	—	—
32	ASA + TP + LMWH + KS	[68, 69]	2	13,816	—	—
33	ASA + PA + TP + UFH + FS	[17]	1	829	—	—
34	KS	[70, 71]	2	6,211	—	—
35	UFH + KS	[65, 70]	2	1,488	—	—

ASA: acetylsalicyclic acid, PA: primary angioplasty, TP: thienopyridines, UFH: unfractionated heparin, LMWH: low molecular weight heparin, GPI: glycoprotein IIbIIIa inhibitors, DTI: direct thrombin inhibitors, FS: fibrin specific thrombolytics and KS: kinase type thrombolytics (streptokinase and urokinase).

**Table 2:** Bleeding criteria found in studies and number of arms that consider each of them.

Number	Criteria	Number (%) of arms
1	TIMI [72] criteria	28 (21.1%)
2	GUSTO [60] criteria	24 (18.0%)
3	Intraocular, retroperitoneal bleeding, transfusion or drop of 50 g/L Hb	2 (1.5%)
4	Fatal bleeding or bleeding requiring transfusion	2 (1.5%)
5	GUSTO [60] criteria, excluding hemorrhagic stroke	11 (8.3%)
6	Fatal bleeding, transfusion of 2 U, drop of 3 g/dL Hb, retroperitoneal, intracranial, ocular or requiring intervention	2 (1.5%)
7	Need for any transfusion	25 (18.8%)
8	Hemorrhagic stroke or transfusion of 2 U	2 (1.5%)
9	Fatal or life threatening bleeding, requiring intervention, prolonged hospitalization, with significant systemic dysfunction	4 (3.0%)
10	Transfusion of at least 2 U, fatal or intracranial bleeding	2 (1.5%)
11	Fall in Hb of at least 2 mmol/L, transfusion of 2 U, need for intervention or intracranial bleeding	2 (1.5%)
12	Transfusion or bleeding excluding puncture site, subcutaneous, hematuria or any drop in Hb described as not serious	2 (1.5%)
13	Severe bleeding of the gastrointestinal tract, resulting in shock or transfusion, critical bleeding and hemorrhagic stroke	2 (1.5%)
14	Nonminor transfusion and bleeding (except puncture site bleeding, streaks of blood in feces and vomit, epistaxis, etc.)	3 (2.3%)
15	Transfusion of at least 2 U	8 (6.0%)
16	Hematemesis, melena, hematuria, and severe hemoptysis	2 (1.5%)
17	Hemorrhagic stroke, hematemesis, severe hematuria, prolonged bleeding, and large hematoma at puncture site	2 (1.5%)
18	Criteria: gastrointestinal and retroperitoneal bleeding, hemorrhagic stroke	4 (3.0%)
19	Hemorrhagic stroke	6 (4.5%)

Hb: hemoglobin; U: international unit of red blood cells—300 mL; CVA: cerebrovascular accident/stroke.

**Table 3:** Multivariate regression with binomial response for death, acute myocardial infarction and major bleeding, with number of drugs (N drugs) and primary angioplasty (PA) as independent variables.

Outcome	Death			Major bleeding			AMI		
Parameter	*β*-Coefficient	Standard error	*P *	*β*-Coefficient	Standard error	*P *	*β*-Coefficient	Standard error	*P *
N drugs	−0.233	0.054	<0.001*	0.405	0.154	0.012*	−0.039	0.064	0.550
PA	−1.605	0.409	<0.001*	2.251	1.049	0.039*	−0.629	0.478	0.197
Interaction1	0.294^a^	0.120	0.019*	−0.355^a^	0.308	0.258	−0.162^a^	0.142	0.262
Latent variable	0.079	0.022	0.001*	0.670	0.176	<0.001*	0.111	0.033	0.002*

	Odds	IC 95%		Odds	IC 95%		Odds	IC 95%	

PA −	0,791	0,704–0,879		1,499	1,030–1,968		0,961	0,836–1,088	
PA +	1,062	0,831–1,293		1,051	0,480–1,622		1,131	0,840–1,422	

Latent variable inserted in the model to minimize the variability effect between studies. Interactions tested in models: ^a^drugs: PA; ^b^PA: bleeding and ^c^PA: death, (without statistical significance). IC 95%: reliability interval 95%.

*P < 0.05.
